# NMR-Based Approaches in the Study of Foods

**DOI:** 10.3390/molecules27227906

**Published:** 2022-11-15

**Authors:** Anatoly P. Sobolev, Cinzia Ingallina, Mattia Spano, Giacomo Di Matteo, Luisa Mannina

**Affiliations:** 1 Magnetic Resonance Laboratory “Segre-Capitani”, Institute for Biological Systems, CNR, Via Salaria, Km 29.300, 00015 Monterotondo, Italy; 2 Laboratory of Food Chemistry, Department of Chemistry and Technology of Drugs, Sapienza University of Rome, P.le Aldo Moro 5, 00185 Rome, Italy

**Keywords:** NMR methodology, food structure, food composition

## Abstract

In this review, the three different NMR-based approaches usually used to study foodstuffs are described, reporting specific examples. The first approach starts with the food of interest that can be investigated using different complementary NMR methodologies to obtain a comprehensive picture of food composition and structure; another approach starts with the specific problem related to a given food (frauds, safety, traceability, geographical and botanical origin, farming methods, food processing, maturation and ageing, etc.) that can be addressed by choosing the most suitable NMR methodology; finally, it is possible to start from a single NMR methodology, developing a broad range of applications to tackle common food-related challenges and different aspects related to foods.

## 1. Introduction

The study of food structure and chemical composition requires adequate analytical methods that have to be comprehensive and as unintrusive as possible. These criteria are largely satisfied by the combination of different NMR methodologies that represents a powerful tool for the study of food matrixes, being recognized as one of the best performing approaches in food analysis [1].

In this review, we want to show how the topic of food can be addressed using NMR methodologies. In the literature, a number of reviews regarding NMR and foods have been already reported. Some reviews relate to specific foods such as wine [2] or honey [3]; others are focused on the application of specific NMR methodologies for food investigation such as HR-MAS [4], NMR relaxometry/MRI [5], and high-resolution NMR in liquids [6,7]. Additional methodologies deal with common food-related challenges such as food frauds [8], authentication, and quality [9]. Taking into account the complexity and richness of this scenario, we tried to aggregate the different approaches into three principal starting points usually used to investigate foods. These three starting points reflect the often converging but sometimes different interests of food producers, retailers, consumers, and researchers.

One starting point focuses on the food that needs to be analyzed. Farmers, companies, and consumers are interested in getting to know the product in depth in order to improve cultivation conditions and processes and for safety reasons. In this case, the combination of different NMR methodologies can be useful to describe different aspects of the product.

Another approach starts from common challenges regarding foods such as frauds, geographical and botanical origins, etc. In this case, consumers and specific research programs constantly stimulate the world of research to propose new solutions.

Finally, it is possible to start from a single specific NMR methodology, developing a broad range of applications to tackle common food-related challenges and different aspects.

These three starting points make use of the information that can be obtained from the different NMR methodologies, namely high-resolution NMR of liquid samples, high-resolution magic-angle spinning (HR-MAS) of semisolid samples, cross-polarization magic-angle spinning (CP-MAS) of solids, low-field relaxometry, and magnetic resonance imaging (MRI). These methodologies can be explained in more detail as follows:

- High-resolution NMR of liquid samples [6] is used to define the chemical structure of isolated compounds or compounds in complex mixtures. It can be applied also to solid and semisolid food samples after extraction of soluble components;
- High-resolution magic-angle spinning (HR-MAS) NMR methodology has been developed to analyze mobile molecular components in all types of matrixes and it is particularly useful in analyzing intact semisolid samples without any extraction. The rotation of the sample around an axis forming an angle of 54.7° (magic angle) with respect to the applied magnetic field B_0_ drastically reduces the broadening of signals due to magnetic susceptibility variations and makes it possible to obtain high-resolution spectra of all mobile molecular components;
- Cross-polarization magic-angle spinning (CP-MAS) is the most frequently used methodology for the NMR analysis of solids. It combines three techniques: fast magic-angle rotation of the sample, high-power decoupling of protons, and cross-polarization (transferring part of the hydrogen magnetization to a heteronucleus to improve its sensitivity) [10]. Generally, in food samples, CP-MAS is applied to study the insoluble fraction (fibers, proteins, etc.), observing ^13^C as the heteronucleus. Moreover, ^31^P and ^2^H solid-state NMR spectroscopic techniques were largely employed to examine the interaction among lipids and many bioactive compounds from foods [11,12,13,14];
- Low-field ^1^H NMR relaxometry [15] is a suitable tool to study the most abundant components of intact foodstuffs by measurement of relaxation parameters (longitudinal relaxation time, T_1_, water self-diffusion (D_w_), T_2_) and amplitudes of the NMR signal. Information on food microstructure such as water compartments and diffusion can be obtained by detecting proton signals dominated by H_2_O contained in foodstuffs. Sample pretreatment is not required, and the method can be easily used for quality control applications;
- MRI allows one to obtain detailed morphological images of internal sections of intact tissues [16]. Furthermore, using specific data acquisition protocols, it is able to provide multiple pieces of complementary information also relating to the composition and dynamics of the molecules that form the investigated tissue.

## 2. First Starting Point: Food

The three different starting points usually used to study foodstuffs by means of NMR methodologies are discussed here, reporting specific examples.

This approach starts with the food that needs to be investigated. It is possible to describe specific and complementary aspects of the product using different NMR methodologies. The chemical-nutritional composition of the investigated food is obtained by means of NMR in the liquid state, HRMAS, and CPMAS methodologies. Using different 1D and 2D sequences, it is possible to obtain the chemical profile of the food or the food extracts. On the other hand, morphological and physico-chemical aspects of the food can be investigated by means of low-field ^1^H NMR relaxometry and MRI.

In the case of vegetables/fruits, the chemical profile and the morphological aspects have often been investigated to obtain information regarding fruit quality and nutritional properties and to monitor fruit changes over the time.

The chemical-nutritional profile of vegetables and fruits is strongly related to their harvesting and post-harvesting time-depending changes. Therefore, the monitoring of metabolite profiling over time allows information on the chemical composition of the samples in specific periods to be obtained. This type of investigation also enables one to detect compounds present only in specific stages of development. This information can be useful in choosing the best harvesting period for direct sales or conservation of the product, but also for obtaining specific nutrients and nutraceuticals.

Ripeness, post-harvesting changes, and shelf-life evaluation can be also investigated by means of MRI and low-field relaxometry that provide complementary information related to the composition and dynamics of the molecules that form the food tissue.

In the case of other products, other aspects can be of interest. For instance, in the case of processed food, the different process phases as well as product storage can be monitored using the most appropriate NMR methodologies.

Here, the case study of kiwifruit is reported. Kiwifruit is a fruit belonging to the family of Actinidiaceae (*Actinidia chinensis* and *Actinidia deliciosa*) native to China. It grows spontaneously in humid environments and is characterized by peculiar flavor, fragrance, and healthful properties. Today, New Zealand and Italy are respectively the first and second largest producers of kiwifruit in the world.

### 2.1. Kiwifruit: Chemical Profile and Morphological Aspects

The chemical profile of kiwifruit’s aqueous and organic extracts has been determined using 1D and 2D high-resolution NMR experiments [17]. Sugars (glucose, fructose, sucrose, myo-inositol, galactose, xylose, raffinose, and mannose), organic acids (malic, citric, quinic, ascorbic, lactic, and shikimic acids), amino acids (alanine, glutamine, threonine, arginine, glutamate, aspartate, valine, leucine, isoleucine, lysine, tryptophane, γ-aminobutyrate, histidine, and phenylalanine), and other compounds (choline, adenosine triphosphate, O^3^-β-d-glucopyranosyl-*trans*/*cis*-caffeic acid, uridine, quercetin 3-rhamnoside, and epicatechin) have been identified and quantified in the ^1^H NMR spectra of kiwifruit aqueous extracts. In the ^1^H spectra of kiwifruit organic extract, fatty acids, phospholipids, sterols, and galactolipids have been identified [18].

Information on water content and water distribution in the sample has been obtained by low-field NMR relaxometry and NMR imaging. Three components of T_2_ relaxation time have been detected and associated with protons in the cell walls, in cytoplasm and in extracellular space, and in the vacuole [19]. The internal morphologies of kiwifruit have been investigated by MRI, which provided informative images regarding the spin density distribution of water molecules and the relationship between water and cellular tissues [20].

### 2.2. Kiwifruit Ripeness

#### 2.2.1. Chemical Profile over the Time (Ripeness and Postharvest Period)

The chemical profile has been monitored over the season, typically from June to December, showing characteristic temporal trends of several compounds. For instance, in the case of organic acids, the content of quinic and ascorbic acids decreases over the season, whereas citric and malic acids have shown the highest level in August. It has been also possible to detect the shikimic acid present only in June and July [21].

Regarding sugars (sucrose, fructose, and glucose), a general increment in their content has been observed, reaching the highest value in December. Furthermore, amino acids have shown specific temporal trends: as an example, the level of valine, leucine, and isoleucine—important essential ramified amino acids—and threonine, glutamine, and asparagine have shown the highest value in July and in August.

**Figure 1:**
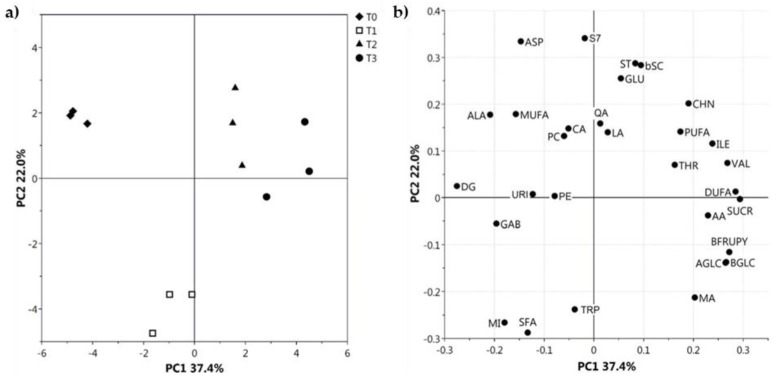
(**a**) PCA score (**b**) and loading plots of metabolite profiles (30 metabolites/metabolite classes) obtained from NMR data related to hydroalcoholic and organic extracts of kiwifruit at different postharvest stages (T0–T3). Results associated with malic acid (MA), citric acid (CA), ascorbic acid (AA), lactic acid (LA), quinic acid (QA), α- and β-glucose (AGLC and BGLC), sucrose (SUCR), β-fructopyranose (BFRUPY), Ala, Thr, Glu, Asp, Val, Ile, Trp and γ-aminobutyric acid (GAB), choline (CHN), uridine (URI), myo-inositol (MI), phosphatidylcholine (PC), phosphatidylethanolamine (PE), digalactosyl diacylglycerol (DG), stigmast-7-en-3β-ol (S7), stigmasterol (ST), poly-unsaturated fatty acids (PUFA), di-unsaturated fatty acids (DUFA), mono-unsaturated fatty acids (MUFA), saturated fatty acids (SFA), and β-sitosterol plus campesterol (bSC) are shown (Reprinted from [18] with permission from Frontiers).

It is well-known that in the case of kiwifruits, before commercialization, a postharvest period is required to obtain the best edible conditions. Different postharvest treatments can be used (cold storage for a long time, exposition to C_2_H_4_, etc.). The chemical profiles of kiwifruits stored in a controlled chamber (at 4 °C, with 85% relative humidity) and removed after 0 (T0), 30 (T1), 60 (T2), and 90 (T3) days of cold storage have been investigated [18]. About 42 metabolites have been identified and quantified in the ^1^H spectra of aqueous extracts (free amino acids, organic acids, sugars, and others) and organic extracts (fatty acids, phospholipids, sterols, and galactolipids). NMR data submitted to chemometric analysis, namely to PCA (Figure 1), provide a general view of metabolite changes associated with postharvest cold storage of fruits at four postharvest stages: from fruit harvest to pre-commercialization. Sugars, ascorbic acid, threonine, isoleucine, valine, and di-unsaturated fatty acids present at the highest level during the final stages of postharvest, whereas alanine and digalactosyl diacylglycerol showed the highest level just after the harvest. Sucrose showed a constant increment during the postharvest period, whereas glucose and fructose increased only during the first stage, remaining relatively constant in the later stages. The level of lactic, quinic, and citric acids remained unchanged in the T0–T3 range, whereas a slight increment in ascorbic acid concentration was observed at the T3 stage. Regarding the organic extracts, a constant increment and a decrement in di-unsaturated fatty acids and digalactosyl diacylglycerol were observed, respectively, whereas the other metabolites remained unchanged.

#### 2.2.2. Morphological Changes over the Time

**Figure 2:**
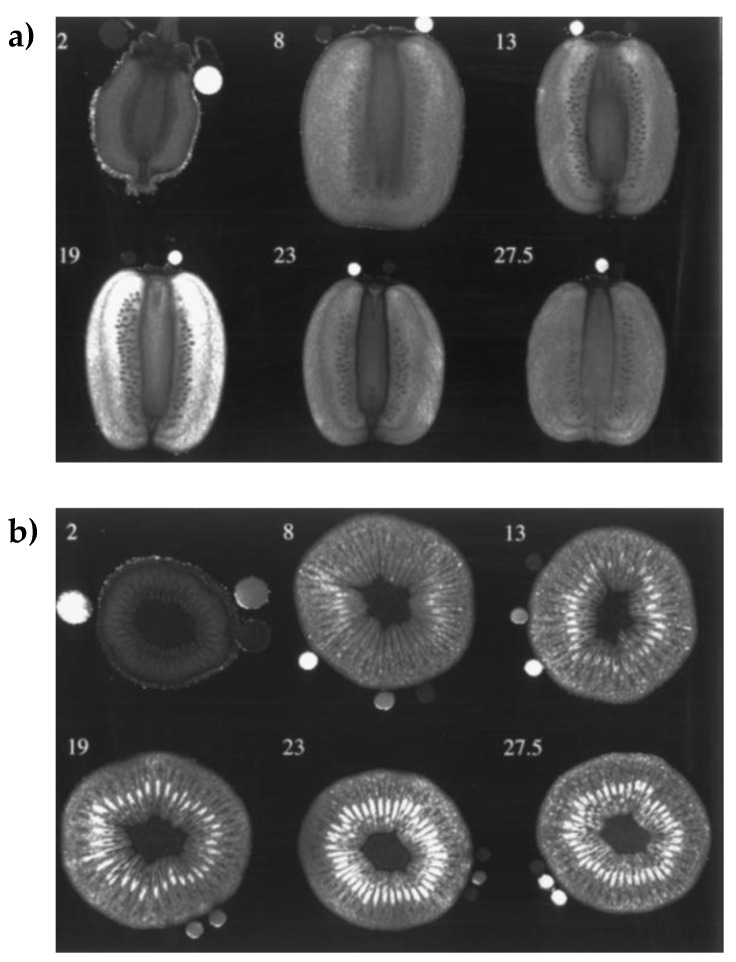
The spin–lattice relaxation (T1) images (**a**) through a longitudinal equatorial plane and spin–spin relaxation (T2) images (**b**) through a transverse equatorial plane of kiwifruit (*A. deliciosa*) during development and ripening. Numbers correspond to weeks from fruitset, with commercial harvest occurring at week 23. Images at 23 and 27.5 weeks are of the same fruit at harvest and after 4.5 weeks ripening. Image resolution varies from 0.12 in the youngest fruit to 0.33 mm in the older samples (256 × 256 pixel data arrays). The circular internal standard tubes (4 mm inner diameter) serve as a scale marker for each panel. Image intensity is based on a continuous grey-scale setting ranging from 0 ms = black to 2500 ms = white (Adapted from [20] with permission from Elsevier, Copyright 1999).

Information regarding the degree of ripeness has been also obtained by means of low-field relaxometry due to the correlation between the changes in T_2_ relaxation time and the increment in kiwifruit “softness” during storage. The results indicate that quantitative MRI is able to measure temporal trends and tissue differences in MR properties of kiwifruit [20]. Measurements were made at a magnetic field strength of 2.0 T (85 MHz). Distinct differences between the relaxation properties of the flesh, locule, and core tissues at particular sampling dates as well as trends during the season (for example, the tendency towards longer relaxation times later in the season in locular tissue) were reported (Figure 2).

**Figure 3:**
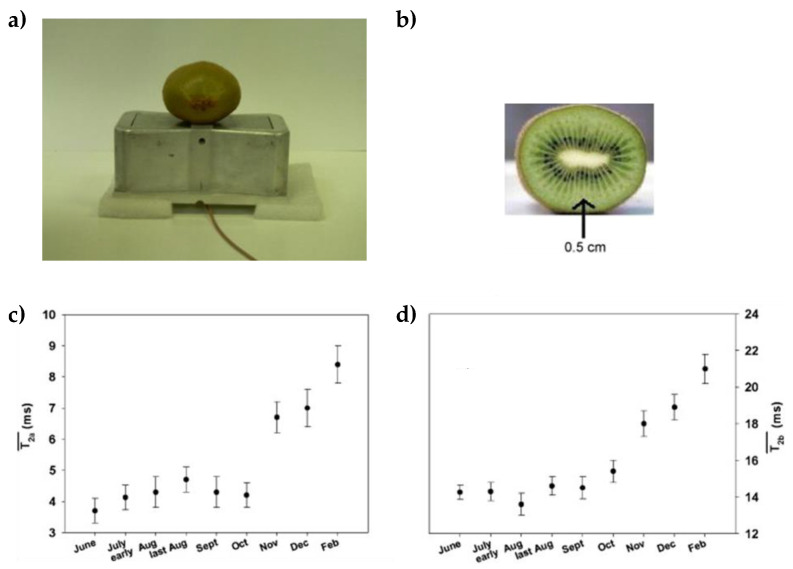
(**a**) Measurement of intact kiwifruit with a portable unilateral NMR instrument. (**b**) Section of kiwifruit showing the depth of measurement with a portable NMR instrument. Average T_2a_ (**c**) and T_2b_ (**d**) were measured on nine kiwifruits vs. the harvesting month; the error bars represent the maximum error calculated with the error propagation theory (Adapted from [17] with permission from Elsevier, Copyright 2010).

In order to study kiwifruit ripeness, T_2_ relaxation times have also been measured directly on the fruit, without cutting it, on pre-harvest kiwifruits by means of a portable single-sided NMR instrument (Figure 3a,b). The experimental condition used to carry out this experiment has allowed only the detection of the two longest T_2_ relaxation times, not detecting the shortest one due to the water signal strictly associated with cell wall compartments. During the ripening phase, an increase in the two T_2_ relaxation times (attributed to water in the cytoplasm and extracellular space, reported as T_2a_, and to water in the vacuole, reported as T_2b_) has been observed. This trend towards longer T_2_ relaxation times over the season reflects a change in the fruit structure (Figure 3 c,d) [17,21].

In kiwifruit, effects of water loss on the water status were investigated using MRI [22], which provides informative images regarding the spin density distribution of water molecules and the relationship between water and cellular tissues. The organization and mobility of water change, thus, induces textural changes and kiwifruit softening. T_2_-weighted images are extremely effective for describing these phenomena.

### 2.3. Comparison among Cultivars

Comparison among the metabolite profiles of different kiwifruit cultivars can be performed. The ^1^H spectra of kiwifruit extracts from different cultivars have shown the same compounds at different concentrations in the same harvesting period. This confirms that every cultivar has a specific time of maturation. For instance, comparison among the ^1^H metabolite profiles of Hayward, CI. GI, and Zespri cultivars at different time points indicates that the maturation of the Zespri cultivar precedes the other two varieties [21].

#### 2.3.1. MRI for Morphological and Physico-Chemical Aspects

MRI high-resolution images are a reliable tool for appreciating morphological differences in fruits. New kiwifruit selections have been investigated by means of MRI: the internal morphologies of these selections have been compared to fruits of the commercial Hayward cultivar [23]. The MRI images show clear morphological differences concerning, for example, the distribution of seeds, the compartmentalization of tissues, and the position of columella. This information can be also useful in suggesting the most promising selections for commercial purposes.

#### 2.3.2. Comparison among Healthy and Non-Healthy Kiwifruit

The metabolite profile has been also useful to discriminate between kiwifruit collected from healthy plants and plants affected by elephantiasis. Elephantiasis causes the progressive degradation of woody tissues and an anomalous diametrical growth of the trunk, making kiwifruits non-compliant with commercial quality standards. The ^1^H NMR spectra of aqueous extract of kiwifruits affected by elephantiasis show a higher number of small oligosaccharides with respect to healthy fruits [24].

## 3. Second Starting Point: Common Food-Related Challenges

The world of food has to face different challenges. Some challenges, such as fraud detection, shelf-life determination, monitoring the geographical origin, etc., are common to many foods, whereas others refer only to specific foods. However, since each food has a specific chemical composition, micro- and macrostructure, texture, etc., the same challenge often requires specific solutions according to the type of food. The NMR methodology has proved to be a sufficiently versatile tool for addressing different specific problems, therefore representing a method of great interest for safeguarding the entire food production chain from manufacturers to consumers.

One of the common food-related challenges of great interest is food fraud detection. Frauds often concern foods of high economic value, which, for example, are substituted or mixed with products of lower economic value, giving a product with poor sensory and nutritional characteristics. Sometimes the fraud may constitute a serious risk to human health and, therefore, it has to be revealed quickly.

Here, some examples of frauds are reported, focusing on cases that cause a change in the chemical composition of the product.

### 3.1. Olive Oils: Addition of Hazelnut Oils to Olive Oils

**Figure 4:**
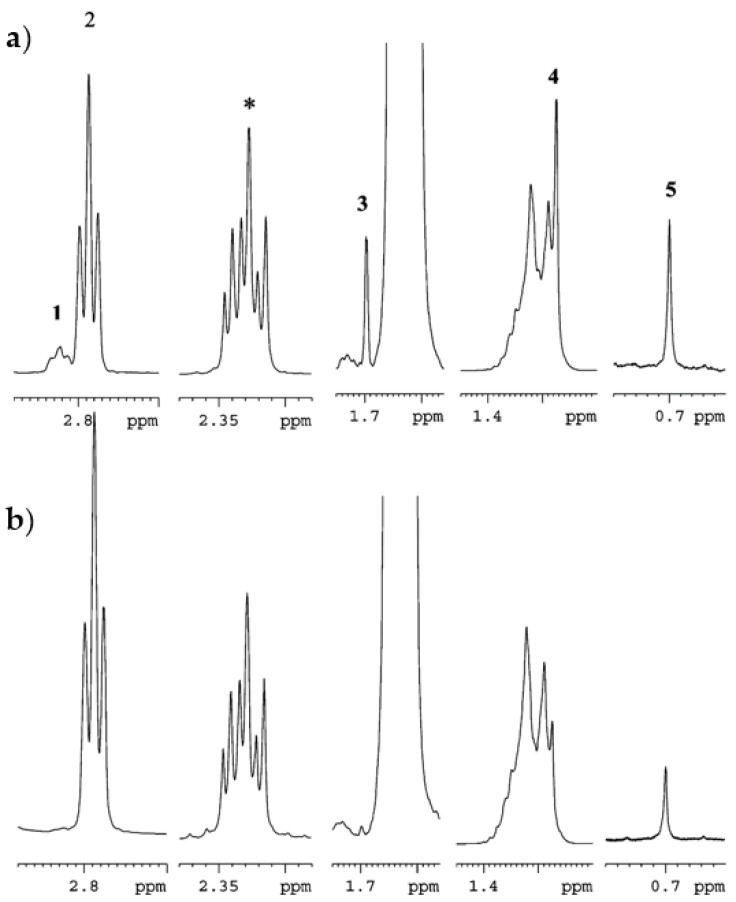
^1^H NMR resonances selected for statistical analyses in the 600.13 MHz ^1^H spectrum of an (**a**) olive oil (top trace) and (**b**) a hazelnut oil. Peaks: 1, diallylic protons of linolenic acid, 2.82 ppm; 2, diallylic protons of linoleic acid, 2.78 ppm; 3, a signal due to squalene, 1.69 ppm; 4, methylenic protons of palmitic and stearic fatty chains, 1.27 ppm; 5, methyl-18 of β-sitosterol, 0.70 ppm. The reference peak at 2.32 ppm is also reported (∗) (Reprinted with permission [26] from American Chemical Society, Copyright 2009).

One of the frauds involving the olive oil sector is the addition of hazelnut oils to olive oils. It is important to clarify that a good-quality hazelnut oil would be more expensive than olive oil: the fraud concerns hazelnut oil made with poor-quality hazelnuts. The composition of the two oils is very similar. However, using the NMR methodology, it is possible to distinguish the two types of oils since, in hazelnut oils, linolenic fatty chains and squalene are almost absent and, therefore, in the ^1^H NMR spectrum, the corresponding signals are almost lacking [25]. On the other hand, the challenge is represented by the difficulty to detect the addition of hazelnut oil to olive oil. A methodology has been proposed: by measuring the intensity of six NMR signals, namely saturated, di-unsaturated, tri-unsaturated fatty chains, squalene, and β-sitosterol, hazelnut oil addition as low as 10% in refined olive oils can be revealed (Figure 4). The ^1^H NMR protocol described according to ISO format has been tested on different NMR instruments operating at different magnetic fields (proton frequency of 600, 500, and 400 MHz) and has been tested by independent laboratories [26]. The issue was also addressed by means of low-field NMR, firstly by Parker et al. [27] and recently by Gouilleux et al. [28,29], demonstrating the efficacy of a profiling strategy based on fast 2D NMR spectra for the detection of hazelnut oil addition into olive oil.

### 3.2. Honey: Fraudulent Designation of Botanical Origin and Addition of Sugars

Honey is a well-appreciated food product. The recognized properties of honey as well as its sensory characteristic depend strongly on the botanical origin. Monofloral honeys have distinct sensory profiles and are more expensive than polyfloral products, giving rise to fraudulent mislabeling and adulterations with honey of lower value or with the addition of sugars. To adulterate honey, different sugary substances have been used: the majority of which are derived from corn, wheat, beets, cane sugar, and rice. One syrup often used as an adulterant is high-fructose corn syrup, known as HFCS. It is a low-cost product and it has a composition of glucose and fructose similar to that of honey. Frauds are more and more frequent and the fraud detection tools available to consumers are few: the problem is open and constantly evolving.

The botanical designation of honey can be put on the label when honey “comes wholly or mainly from that particular source and has the organoleptic, physical-chemical and microscopic properties corresponding with that origin.” [30]. However, clear analytical parameters to define monofloral honey do not exist. To date, melissopalynological analysis is the only technique used to characterize monofloral and polyfloral samples as well as to determine the geographical origin. However, the results of this analysis can be strongly influenced by possible contaminations and the development of suitable analytical methodologies is strongly required.

Among the frauds mentioned before, the NMR methodology has provided an important contribution to the detection of sugars’ addition in honey samples and to the determination of incorrect labeling that does not reflect the exact geographical or botanical origin. The NMR methodology is also useful in determining the presence of adulterants, in quantifying them, and in building high-performance prediction models that can establish the origin and genuineness of commercial samples.

#### 3.2.1. Addition of Sugars in Honey Samples

The aqueous ^1^H spectra of honey samples are dominated by strong signals of sugars [31]. The ^1^H spectrum of honey in DMSO-d_6_ reveals the presence of exogenous sugars (up to 10% by weight), through the identification of hydroxyl groups of maltose, sucrose, and other minority oligosaccharides, completely absent or present only in very small amounts in unadulterated samples. Therefore, in the adulterated honey samples, groups of signals due generally to maltose and other polysaccharides absent in the genuine product are clearly visible. In some cases, the adulteration is more sophisticated and it is necessary to determine the complete sugar profile of the investigated product and compare the profile with that of authentic honey. The saccharides identified in the ^1^H spectrum can be divided into three categories: endogenous sugars typical of honey, not detected or present only in traces in commercial sugar syrups; exogenous sugars present only in syrups; and sugars determined both in authentic honey and in some commercial sugar syrups. The sugar profile has been investigated in the case of acacia honey. The concentration range of endogenous sugars in genuine European acacia honey has been taken as a reference to evaluate the authenticity of a sample of the same botanical origin. Concentration levels significantly lower than the reference values could be indicative of adulteration of the honey by, for instance, dilution with sugar syrup. On the other hand, the presence of exogenous saccharides is strong evidence of adulteration.

**Figure 5:**
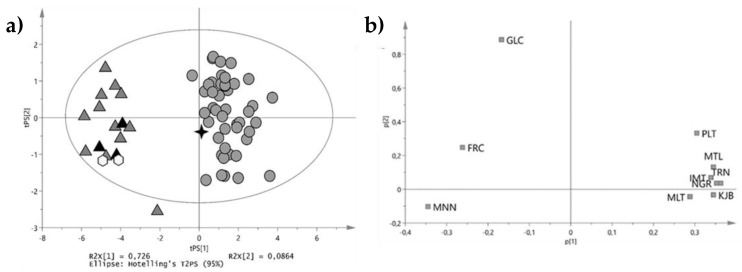
(**a**) Score plot of PCA performed on the sugars matrix. Sucrose and erlose were not considered in this analysis because of their large concentration variability. PC1 and PC2 accounts for 81% of the total variance (i.e., 72.6% + 8.64%). Grey circles: European honey; grey triangles: commercial Chinese honey; black triangles: Chinese honey analyzed with SCIRA; black star: Chinese honey purchased in China; white hexagons: honey from bee-feeding experiments. (**b**) Loading plot that highlights the metabolites responsible for the cluster separation (Reprinted from [32], with permission from Elsevier, Copyright 2020).

A comparison between European and Chinese acacia honey samples has been reported [32]. In this case, principal component analysis (PCA) has been applied: samples were divided into two clusters as shown by the score plot (Figure 5). Chinese honey samples were characterized by a higher content of monosaccharides (glucose, fructose, and mannose) and a lower content of oligosaccharides. Several Chinese honey samples circulating in the European market showed a relevant presence of mannose, absent in genuine acacia honeys and a lower content of endogenous disaccharides typical for European ones. Mannose is instead present in rice syrups and in those with a high fructose content. The sugar NMR profile of a certainly genuine acacia sample from China falls within that of European honeys; this makes us assume that the different values do not depend on geographical origin but on adulteration. It is important to underline that both the melissopalynological analysis and the EA/LC–IRMS did not reveal particular anomalies in these Chinese samples, confirming their declared botanical origin. This further underlines the importance of NMR analysis and the great potential of the CSSF–TOCSY method in validating the authenticity of honey [32].

#### 3.2.2. Fraudulent Designation of Botanical Origin

The NMR methodology addresses the problem of honey’s botanical origin by proposing various solutions. Here, only a few significant examples are reported. One NMR approach is based on the observation that organic honey extracts of different botanical origins show a distinct chemical profile and, in several cases, some molecules can be considered as specific markers, being present only in specific honey samples [33,34]. Here, the markers identified in the ^1^H spectra of several honey samples (organic extracts) are reported:

Linden honey:

- 4-(1-hydroxy-1-methylethyl)cyclohexa-1,3-diencarboxyl methyl ester
- 4-(1-hydroxy-1-methylethyl)cyclohexa-1,3-dienecarboxylic acid
- 4-(1-methylethenyl)cyclohexa-1,3-diencarboxylic acid
- 4-(1-hydroxy-1-methylethyl) benzoic acid
- (E)-2,6-dimethyl-3,7-octadiene-2,6-diol;

Chestnut honey:

- deoxyvasicinone
- γ-lactam derivative of 3-(2′-pyrrolidinyl)-kinurenic acid
- γ-LACT-3-PKA
- 2-quinolone
- 4-quinolone;

Acacia honey:

- pinocembrin
- chrysin
- alpinone
- (Z, E)-abscissic acid;

Orange honey:

- caffeine
- (E)-2,6-dimethylotta-2,7-dien-1,6-diol (8-hydroxylinalol);

Eucalyptus honey:

- dehydrovomifoliol
- 3-oxo-α-ionone;

Strawberry tree honey:

- unedone homogenistic acid.

**Figure 6:**
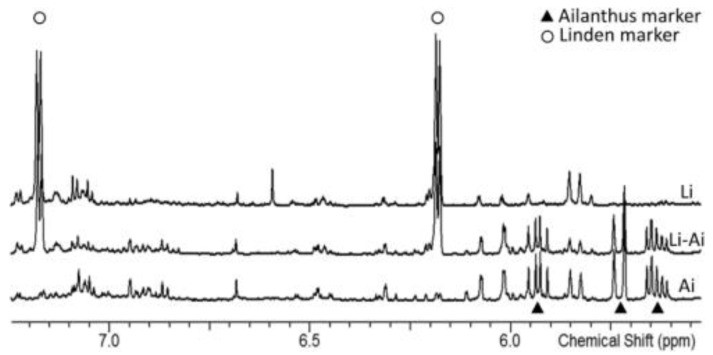
A region of the ^1^H NMR spectra of chloroform extracts where the linden and ailanthus markers resonate: (from top to bottom) monofloral linden honey; honey containing both linden and ailanthus; monofloral ailanthus honey (Reprinted with permission from [35]. Copyright 2016 American Chemical Society).

In some easy cases, it is possible to identify the co-presence of several botanical species in the same honey samples thanks to the presence in the ^1^H spectra of the signals of the specific markers. For instance, the ^1^H spectrum of honey samples from two botanical species, namely ailanthus and linden, clearly shows the presence of the signals due to specific markers of the two botanical species, as shown in Figure 6 [35].

However, it is possible to build classification models considering each type of honey as a single class. To identify the different floral contributions in honey, a statistical approach that considers all types of honey has been developed. This approach is based on *n* models of the OPLS-DA type (one for each botanical type), in which each botanical type is compared with all the others. It allows us to highlight the floral contributions in a given honey [36]. For instance, if you have 20 different flower varieties, 20 models called “one vs. all” are built. Given an unknown honey, each model returns the probability of belonging to a certain type. In this way, it is possible to identify the main floral contributions in the honey.

### 3.3. Coffee

#### 3.3.1. Fraudulent Addition of Robusta to Arabica Coffee

**Figure 7:**
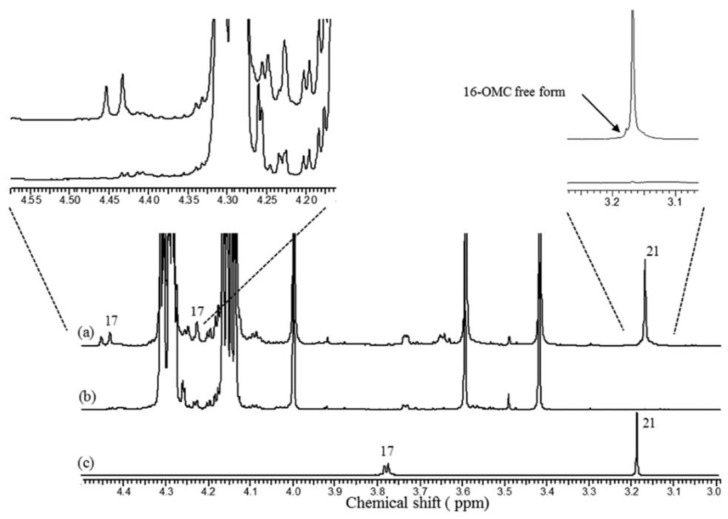
Expanded region of the ^1^H NMR spectrum showing the diagnostic resonances of protons 17 and 21: (**a**) 100% *Robusta* sample, (**b**) 100% Arabica sample, and (**c**) 16-OMC standard. In the insets on the top of the figure, the signals of protons 17 and 21 of 16-OMC ester are enlarged. They are clearly visible in the *Robusta* sample (top), but they are absent from the Arabica sample. The arrow shows the weak methyl signals in position 21 of 16-OMC in its free form (Adapted with permission from [39]. Copyright 2014, American Chemical Society).

Coffee from Arabica species is the most valuable and expensive type of coffee. The problem of adulteration of Arabica coffee with the less valuable Robusta coffee can be addressed by measuring the amount of specific fat-soluble markers belonging to the class of diterpenes. Kahweol is characteristic of the Arabica variety and is present only in trace amounts in Robusta coffee samples; on the other hand, 16-O-methylcafestol is present only in Robusta coffee. To date, the official method for authenticity testing is the German standard method DIN 10,779 [37], which provides the quantification of 16-O-methylcafestol in coffee through a lipid extraction and a high-performance liquid chromatography (HPLC) analysis. The marker of Robusta coffee, 16-O-methylcafestol, can be detected in the ^1^H NMR spectrum of the chloroform extract of the roasted coffee powder thanks to the characteristic signal of the methoxide group at 3.17 ppm, as shown in Figure 7 [38,39]. In this way, by means of ^1^H NMR, the presence of Robusta coffee traces (with an approximate limit of detection of 1–3%) in Arabica coffee is detectable. However, a common problem of the method that exploits the 16-O-methylcafestol is its wide range of concentration in different Robusta samples, which acts as an obstacle to the quantification of Robusta percentage in roasted coffee blends. Another issue regarding the coffee discrimination based only on the 16-O-methylcafestol quantification was reported by Gunning et al. [40], who found a small marker peak at 3.17 ppm in 30 Arabica coffees of assured origin. They have also established that the investigated signal arises not only from 16-O-methylcafestol, but likely from both 16-O-methylcafestol and 16-O-methylkahweol. The article concludes that the usage of other methods may not need to be affected if their detection limit for 16-O-methylcafestol is above the levels observed in authentic Arabica coffees, as appears to be the case for the DIN official method. However, regarding NMR application in the coffee fraud issue, the 3.17 ppm peak area can be used as a reasonable proxy for the Robusta content in mixture samples by defining a threshold, marking the upper limit of the normal Arabica range. Moreover, the researchers have established the possibility of using a 60 MHz low-field NMR method to assess the coffee’s authenticity, since for the quantification of a single, well-resolved spectral peak, there is no advantage in using the higher-field-strength NMR. Other studies regarding the discrimination of Arabica and Robusta coffee were focused on the aqueous extract and take into account the entire metabolic profile [41]. In this case, the NMR spectra were used as input to create an OPLS model, using the different metabolite amounts of the two cultivars. This approach could prevent frauds and erroneous evaluations, providing discrimination that takes into account several chemical compounds simultaneously instead of a single marker.

#### 3.3.2. Geographical Origin

Another issue regarding coffee chain production addressed by means of NMR is the determination of the geographical origin from the perspective of fraud detection. Presently, the geographical origin of coffee is not determined analytically, whereas NMR spectroscopy combined with chemometrics resulted in a powerful and objective tool to trace the authenticity of roasted coffee. This kind of determination could be very useful for the trade sectors that require a high level of traceability to ensure quality and geographical origin. Here, some significant examples are reported. One NMR approach has been based on the discrimination of aqueous extracts of roasted coffee from three main production areas: America, Africa, and Asia [42]. The resulting NMR data analyzed with an OPLS-DA model indicated that American roasted coffee samples are characterized by a higher level of fatty acid chains, the African samples contain more chlorogenic acids and lactate, and the Asian ones are characterized by a high content of acetate and trigonelline, leading to a clear discrimination of roasted coffee samples according to their continent of origin. In another study, coffee samples (methanol coffee extracts) from Colombia, one of the main producers of coffee in the world, have been well-distinguished with respect to the coffee of other countries, including Colombia’s closest neighbors, using NMR fingerprinting combined with chemometric analysis, namely PLS-DA [43]. Discrimination of coffees from Colombia is based mainly on a different content of fatty acids, acetate, and caffeine. This approach could represent a relevant opportunity to protect national productions.

A further NMR study has investigated the effect of different manufacturing process on the chemical composition of coffee. NMR data regarding instant coffee aqueous extracts (soluble extracts prepared industrially from coffee blends of different origins and varieties) from three different producers have been subjected to principal component analysis (PCA) followed by linear discriminant analysis (LDA). Coffee samples have been grouped according to their manufacturer, suggesting that NMR analysis can be useful to monitor the reproducibility and variations in industrial products. Moreover, it is possible to monitor coffee authenticity and quality assurance, allowing the discovery of possible frauds due to the sale of low-quality products marked as high-quality ones [44].

### 3.4. Saffron

Saffron is one of the most used spices in the world and is well-known for its characteristic color, aroma, and flavor, respectively owing to the presence of crocins, safranal, and picrocrocin [45]. The collection and production of this spice are very laborious processes and, considering that the number of stigmas per flower is very low, a great number of flowers are needed—about 450,000—to obtain 1 kg of product. All these aspects have made saffron a very expensive spice and, consequently, this product is subjected to several fraud actions, represented by the addiction of plant-derived materials *(C. sativus* stamens, arnica, calendula, cayenne pepper, sandalwood, safflower, turmeric, and gardenia) or artificial colorants as bulking agents [46]. High-resolution ^1^H NMR combined with chemometrics has shown to be very effective in tracing this kind of fraud by developing predictive models able to discriminate original saffron from adulterated samples with a minimum of 20% *w/w* bulking agents [47,48]. In a recent study, an improvement of this sensitivity has been obtained, detecting a 5% *w*/*w* saffron adulteration with safflower and turmeric [49]. In particular, the application of both unsupervised (PCA, HCD) and supervised (PLS-DA) chemometric models on ^1^H NMR spectra of pure and adulterated saffron aqueous extracts allowed the detection of statistically significant variability, mainly due to sugars (positively related to adulterated samples), crocin, and formic acid (positively related to pure saffron). The application of the same protocol on ^1^H NMR spectra of DMSO-*d_6_* organic extracts resulted in a discrimination between pure and adulterated saffron samples mainly due to curcuminoids (positively related to turmeric-adulterated saffron), linoleic acid (positively related to safflower-adulterated saffron), crocins, and safranal (positively related to pure saffron).

**Figure 8:**
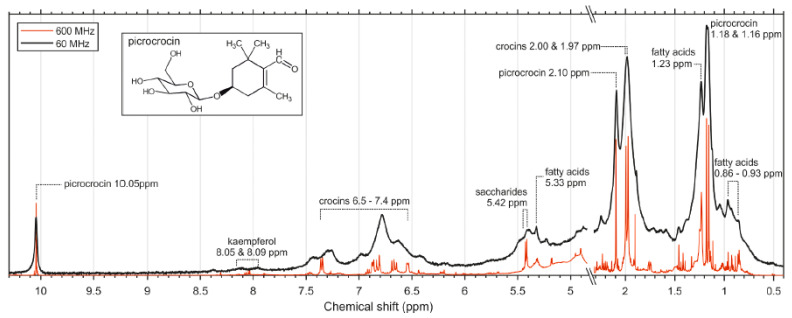
Comparison of 60 MHz (benchtop) and 600 MHz (high-field) ^1^H NMR spectra obtained from an extract of saffron in DMSO-d6, with annotations of the main features identified in the 60 MHz spectrum. The benchtop spectral profile is dominated by resonances attributed to picrocrocin, a major saffron metabolite, the structure of which is given in the inset panel. The isolated peak at 10.05 ppm is in a generally uncrowded region of the spectrum and provides a useful indicator of the presence of saffron in a sample. Reprinted from [50], with permission from Elsevier, Copyright 2020.

Recently, saffron adulterations with arnica, calendula, safflower, turmeric, cayenne pepper, sandalwood, and tartrazine have been analyzed using benchtop 60 MHz NMR analysis proposed as an alternative to the high-field NMR approach [50]. In particular, the benchtop 60 MHz NMR analysis of DMSO-*d_6_* organic extracts of pure saffron has been able to detect some typical signals of crocins and picrocrocin (Figure 8), making it possible to use these signals as elements of distinction between pure and adulterated saffron.

The analysis of pure and adulterated saffron samples at different bulking percentages (5, 10, 20, 30, 40, 50%) has allowed the detection of, through the application of DD-SIMCA, OCC-NN, and IF one-class classification approaches, statistically significant differences between analyzed samples, although the minimum detectable bulking percentage was 30%. Additionally, this approach can offer a low-cost routine method for authenticity screening.

### 3.5. Milk

Milk is a very complex and nutritionally rich foodstuff whose study through NMR spectroscopy has allowed researchers to solve several issues related to origin, production practices, and adulteration [51,52,53,54]. Here, two representative examples related to milk adulteration detected by applying different NMR approaches are discussed. In the first study, high-resolution ^1^H NMR spectroscopy has been applied to detect adulteration of caprine milk with cheaper bovine milk [55]. In particular, the application of PCA and OPLS-DA chemometrics approaches on ^1^H binned spectra of aqueous extract of pure and adulterated (1, 5, and 10%) caprine milk has facilitated predictive models able to detect this kind of adulteration through the identification of specific markers, namely N-acetyl carbohydrates, typical of bovine milk.

Interestingly, ^1^H TD-NMR has also been successfully applied to detect milk adulterations in a study where bovine milk was spiked with known levels (from 5 to 50% *v/v*) of whey, urea, hydrogen peroxide, synthetic urine, and synthetic milk [56]. In particular, T_2_ relaxation time was significantly affected by adulteration, showing, with respect to the T_2_ value of pure milk, an increase in its value depending on the adulterant percentage. This significant difference has also been confirmed by exploratory analysis (PCA), classification (SIMCA and kNN), and proposition of regression models (PLSR), thus confirming the potential use of ^1^H TD-NMR as an alternative rapid method for detecting and quantifying milk adulteration.

## 4. Third Starting Point: NMR Methodology

**Scheme 1:**
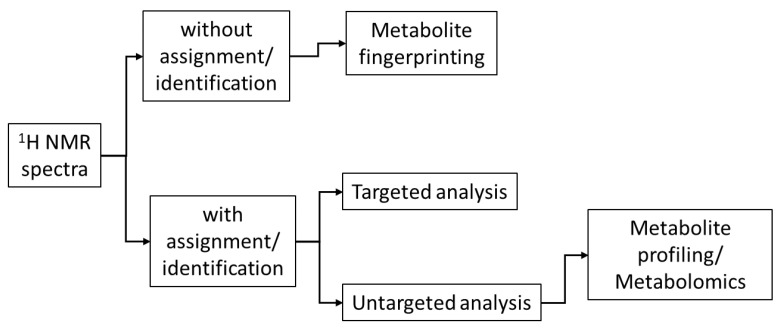
Classification of high-resolution ^1^H NMR spectroscopy’s application types for food analysis.

As previously reported, each NMR methodology can be useful in investigating specific aspects related to foods. In particular, high-resolution ^1^H-NMR spectroscopy in solutions can be applied to the study of foods, following different approaches that can be classified as metabolite fingerprinting, target analysis, and untargeted analysis (Scheme 1).

Both targeted and untargeted analysis required the compounds’ identification. In fact, the NMR methodology is well-known to be suitable for the identification of the chemical structure of compounds in solutions, especially those with relatively low molecular weight (less than 1 kDa). The high resolution of NMR spectra makes it also possible to study complex mixtures, such as liquid foods and food extracts, without physical separation of the components. However, due to the extreme signal overlapping in the spectra of foodstuffs and in order to realize a complete signal assignment, 2D NMR experiments [7], 1D selective pulse experiments [57,58], and the DOSY experiment [59] are usually performed. The 2D experiments give us a plane surface with a series of cross-peaks that represent correlations between two frequencies. The most common 2D experiments are ^1^H-^1^H TOCSY, ^1^H-^1^H COSY, ^1^H-^13^C HSQC, and ^1^H-^13^C HMBC. They are powerful experiments for the identification of all the correlations ^1^H-^1^H and ^1^H-^13^C for each metabolite in complex mixtures in order to realize a complete structure assignment of the majority of the present compounds. Among the various types of 1D selective NMR experiments that are available, one of the most useful is the 1D TOCSY selective pulse sequence, in which a single resonance is selectively irradiated, and the magnetization propagates through the ^1^H coupling network [57]. It is a powerful experiment for distinct spin systems within the molecule, potentially revealing multiple structures that were otherwise overlapped or buried. The DOSY experiment is recorded as a series of 1D NMR spectra with an increasing magnetic field strength gradient, finally combined to obtain a 2D NMR spectrum, showing NMR chemical shifts and self-diffusion coefficients on the horizontal and vertical axes, respectively. The DOSY experiment is particularly useful in the case of mixtures of compounds with different molecular weights. It is also helpful in spiking experiments and databases containing NMR data of metabolites (HMDB, MMCD, TOCCATA) [60,61]. Identification of components can be followed by their quantification (qNMR).

When absolute metabolite quantification is required, the duration of recycle time and the number of scans to reach a suitable signal-to-noise ratio have to be optimized in each experiment. The traditional quantification method is represented by the integration of a specific signal area vs. the signal area of a reference compound. In aqueous solutions, trimethylsilylpropanoic acid sodium salt (TSP) and sodium trimethylsilyl propanesufonate (DSS) have been widely used, whereas hexamethyldisiloxane (HMDSO) has been proposed for organic solutions. An alternative to the use of the internal standard is represented by the Electronic REference To access In vivo Concentrations (ERETIC) and QUANTification by Artificial Signal (QUANTAS) methods that provide a reference signal synthesized by an electronic device. Particular attention must be paid to the integration procedure when the water peak dominates the ^1^H spectrum of a foodstuff sample. In this case, it is difficult to perform a correct quantitative analysis of the signals close to the water signal tail. Moreover, water suppression procedures can also partially saturate the resonances near the water signal, affecting their quantification. So, experimental water suppression parameters must be accurately calibrated. Finally, in another quantification method, the measure of the signal height, reported as signal intensity, is used instead of the integral for quantitative measurements since, as in the case of the integral, signal intensity is proportional to the compound concentration. In order to use this approach, the linewidth of each specific resonance has to be the same in all compared spectra. The measurement of the intensity can be performed manually or, in the case of many signals, by using an automatic peak-peaking procedure. Once the metabolite identification has been realized, the targeted or untargeted approach can be applied.

In the targeted analysis, the identification and quantification of single or a few marker compounds, chosen according to previous knowledge about the food under investigation, are performed. Metabolite extraction and other preparation procedures are carried out in order to concentrate the selected metabolite and to remove interference from other compounds. This approach is mainly applied to identify compounds that are responsible for some peculiar characteristic of the analyzed foodstuff, thus characterizing its origin, variety, or identifying adulterations. An example is the reported case regarding coffee in Section 3.3.1, in which the quantification of only 16-O-methylcafestol [38] or a few characteristic metabolites [62,63] in coffee samples allows the discrimination between Arabica and Robusta coffee varieties and the fraudulent addition of Robusta to Arabica coffee. Another example is the protocol of targeted ^1^H NMR quantitative analysis of six common sugars in food and beverages developed by Yang et al. [64]. Fructose, galactose, glucose, lactose, maltose, and sucrose were simultaneously quantified in a single high-resolution ^1^H NMR experiment. The sample preparation procedure for the analysis was very simple: an aliquot of a liquid sample was diluted in D_2_O followed by direct NMR analysis, whereas solid samples were suspended in H_2_O, then the soluble fraction was filtered, concentrated in vacuum, and dissolved in D_2_O. The quantification procedure was based on the integration areas of the characteristic proton signals. The method was successfully applied to a wide range of food and beverages including black tea, fruit juices, vinegar, wine, sake, soy milk and soy sauce, chocolate, honey, cookie, sauce paste, etc. The targeted NMR approach has been mainly used in food authentication, e.g., to distinguish between the two marketable oregano species with apigenin and p-cymene as biomarkers of *Origanum onites* and salvianolic acid B of *Origanum vulgare* [65]; to identify fraudulent processing techniques (with phlorin as marker) [66] or adulteration (with coumarins and psoralens as markers) [67] of Citrus juices; to evaluate saccharide adulteration in honey [68]; to detect illegal adulterants in herbal medicines [69]; and to quantify compound markers of quality in spirit drinks [70]. The targeted approach has been also used for the quantification of chosen metabolites in food matrices, e.g., in the quantification of polyols in sugar-free foodstuffs by the Chemical Shift Selective Filter–Total Correlation Spectroscopy (CSSF–TOCSY) NMR experiment [71]; the analysis of fatty acids in walnut oil [72]; the quantification of methylglyoxal in manuka honey [73]; and the quantification of cannabinoids in hempseeds [74] and hempseed oils [75]. Targeted analysis with quantification of the two best known cannabinoids (Δ9-tetrahydrocannabinol and cannabidiol) has been also realized by Araneda et al. [76] using a benchtop NMR instrument.

Untargeted analysis consists in the identification and quantification of a number of metabolites belonging to various classes of compounds in a given sample, often without any separation procedure. The untargeted or bottom–up approach does not rely on a predetermined hypothesis and, therefore, the solution to a problem may require the identification and quantification of as many metabolites as possible. All the quantified metabolites are then normally used as input for the chemometric analysis. Here, the case study of sea bass is discussed as an example of untargeted analysis. In this study, a mixed metabolic profiling–metabolomics approach was followed [77]. High-resolution NMR spectroscopy was used as an analytical tool to determine the complete metabolic profiling of sea bass extracts: the ^1^H spectra of sea bass extracts in aqueous and organic solvents were analyzed, all the NMR signals were assigned using 2D NMR spectra, and finally, the content of the identified metabolites was measured and submitted to chemometric analysis. Water-soluble metabolites belonging to different classes such as sugars, amino acids, dipeptides, and organic acids as well as metabolites soluble in organic solvents such as lipids, sterols, and fatty acids were identified. Metabolite profiling and suitable statistical analysis were used to discriminate between wild and cultured sea bass samples. Preliminary results showed that discrimination between wild and cultured sea bass was obtained not only using fatty acid composition, but also cholesterol and phosphatidylethanolamine and some water-soluble metabolites such as choline, trimethylamine oxide, glutamine, and fumaric and malic acids. This type of approach has been frequently used to study various aspects of foods, such as pedoclimatic factors (olive oil [78,79,80]), geographical origin (wines [81,82,83], olive oils [84,85,86]), botanical origin (honey [34,87]), ripening (kiwi [21], tomatoes [88,89], pistachio [90]), postharvest changes (kiwi [18], banana [91]), and the manufacturer (beer [92]). Food authenticity (wine [93,94,95], honey [32,96], coffee [97]) has been also studied with the described untargeted approach.

Unlike the targeted and untargeted approaches, metabolic fingerprinting does not require metabolite identification. In fact, even without identification, an ^1^H NMR spectrum represents a kind of fingerprint, reflecting the metabolic composition of a given foodstuff that can be used as such for traceability monitoring and other purposes. Although identification is preferable, it requires additional time, effort, and costs that are not always justified. In this approach, all the NMR resonances are measured without any identification. Then, the whole spectra (keeping the original number of data points) or the spectra divided in buckets (or bins) are used as input for the chemometric analysis. Metabolite fingerprinting (namely the entire ^1^H NMR spectrum) is used for rapid screening of a set of food samples to compare the samples of different origins, for traceability purposes, and for monitoring the possible changes in the same matrix with time or during a specific process. It is the case of wheat grain flour characterization by ^1^H NMR that aimed to compare three fractions (bran, germ, and refined wheat) and whole-grain flour of two wheat varieties [98]. The entire ^1^H NMR spectra of wheat flour polar (with methanol) and apolar (with chloroform) extracts were acquired and binned to reduce the number of variables (from 32,000 down to 700–90, depending on the size of the binning interval). Multivariate statistics (PCA) of NMR spectrum-derived variables enabled authors to clearly distinguish the three fractions and the whole-grain flour samples. The NMR profiling approach has been largely used to study the same food aspects described for the untargeted NMR analysis: pedoclimatic factors (olive oil [99,100]), geographical origin (wines [101,102], olive oils [103,104,105], cocoa beans [106], coffee [41], saffron [107], asparagus [108], walnuts [109], hazelnut [110], honey [111]), botanical origin (honey [112]), ripening (tomatoes (64), passion fruit (69)), post-harvesting (peaches, tomatoes, and plums [113]), and the manufacturer (beer [114], coffee [44]). Food authenticity (seeds [115], olive oil [116], beer [117], honey [118], avocado oil [119]) has been also studied with the described untargeted approach.

It is also common to combine the NMR profiling approach with the untargeted approach: the whole NMR spectra or bins (buckets) of the spectra are used as input for the chemometric analysis, and then the identification of some signals of interest is performed. However, both untargeted NMR analysis and metabolite fingerprinting generate a large amount of data from which it is necessary to extract the desired information using chemometric methods that can identify possible patterns among samples. In fact, since the number of identified metabolites and the number of studied samples can be very consistent, the only possibility of generating some final consideration or hypothesis from the obtained data is represented by the application of proper statistical approaches. The nature of quantitative data extracted from an NMR spectrum influences the choice of the statistical approach for their elaboration. Two types of statistical approaches can be used for data analysis, namely univariate and multivariate [120]. Among these, multivariate statistical analysis is generally applied for processing NMR data from complex matrices such as foodstuffs by means of unsupervised and supervised approaches. Explorative unsupervised analysis such as principal component analysis (PCA) and tree clustering analysis (TCA) is often the first step of statistical treatment. The structure of data, presence of outliers, and natural grouping of samples can be revealed at this step and in many cases, the obtained results are sufficient to distinguish different groups of samples according to metabolic differences. However, in the case of classification and prediction problems, the application of supervised multivariate techniques (PLS, LDA, PLS-DA, OPLS-DA, SIMCA) is necessary.

To date, together with these conventional statistic approaches, machine learning and neural networks are applied to gain useful insights and a corresponding interpretation of NMR outcomes [121,122]. Machine learning is a branch of artificial intelligence that is involved in finding links among complex patterns of molecular data through classification, clustering, features discovery, and integration [123]. Machine learning applied to NMR data is a useful tool for the dimensional reduction of the spectral data and the classification and clustering of the spectral data in latent feature spaces. Machine learning has been successfully applied to solve many food issues, including fraud detection, geographical origin, production/processing procedures, and food safety [122].

## 5. Conclusions

The three starting points reported suggest that the combination of food-related challenges and NMR methodologies’ capacity to cope with them still has infinite possibilities to be explored. All three approaches will benefit from the development of the NMR sector, which includes advancements in methodologies and techniques (new NMR pulse sequences, new instrumentation, etc.). New trends concern the automation of NMR analysis to exclude human error and lower the analysis cost, making this methodology more accessible for widespread applications in industry and in food control companies. Until a few years ago, the technological trend in NMR-related instrumentation was to go towards higher magnetic fields to increase the sensitivity and spectral resolution. Now, in parallel, there is a growing tendency to obtain better performances for spectrometers operating at medium and low magnetic fields generated by permanent magnets instead of superconductive ones [124,125,126]. Since, unlike superconductive magnets, permanent magnets do not need costly cryogenic liquids (liquid helium and nitrogen) for maintenance, lower costs make them more suitable for extended use in companies and research centers. So, the idea is to adapt the solutions found with high-field NMR spectrometers to low-field ones.

A further aspect of great interest to the NMR community is the unification of the analytical protocols used to investigate foods. Many foods have been analyzed using different experimental methods (extraction procedures, solvents, magnetic fields, etc.). This lack of uniformity often yields results that cannot be compared and, in any case, cannot be proposed as official methods. A great effort expected from the NMR scientific community is, therefore, to standardize specific analytical procedures for each food.

Finally, the scientific community is apt to create a food NMR database sharable not only among the scientific world but also with companies to provide an in-depth knowledge of the products.

## Data Availability

Not applicable.
